# 4,6-Dimeth­oxy­pyrimidin-2-amine–2-(1*H*-indol-3-yl)acetic acid (1/1)

**DOI:** 10.1107/S1600536810037724

**Published:** 2010-09-25

**Authors:** Samuel Ebenezer, Packianathan Thomas Muthiah

**Affiliations:** a School of Chemistry, Bharathidasan University, Tiruchirappalli-620 024, Tamilnadu, India.

## Abstract

In the title co-crystal C_6_H_9_N_3_O_2_·C_10_H_9_NO_2_, the 4,6-dimeth­oxy­pyrimidin-2-amine mol­ecule inter­acts with the carboxyl group of the 2-(1*H*-indol-3-yl)acetic acid mol­ecule through N—H⋯O and O—H⋯N hydrogen bonds, forming a cyclic hydrogen-bonded *R*_2_^2^(8) motif, which is further linked by an N—H⋯N hydrogen bond, forming a supra­molecular chain along the *c* axis. Neighboring chains are inter­linked *via* C—H⋯O hydrogen bonds, forming a supra­molecular ladder

## Related literature

For background to crystal engineering, see: Desiraju (1989 ▶). For the role of amino­pyrimidine–carboxyl­ate inter­actions in protein-nuleic acid recognition and protein-drug binding, see: Hunt *et al.* (1980 ▶); Baker & Santi (1965 ▶). 2-Amino­pyrimidine forms a wide variety of 1:1 adducts with mono and dicarb­oxy­lic acids (Etter & Adsmond, 1990 ▶) rather than individual self-assembly compounds (Scheinbeim & Schempp, 1976 ▶). The 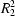
(8) motif is frequently observed when a carb­oxy­lic acid inter­acts with a 2-amino heterocyclic ring system, see: Lynch & Jones (2004 ▶). It is also one of the most commonly occuring motifs, see: Allen *et al.* (1998 ▶). For the biological activity of amino­pyrimidine derivatives and 2-(1*H*-indol-3-yl)acetic acid, see: Hunt *et al.* (1980 ▶); Arteca (1996 ▶). For related structures, see: Karle *et al.* (1964 ▶); Low *et al.* (2002 ▶). For related co-crystals of amino­pyrimidines, see: Thanigaimani *et al.* (2006 ▶, 2007 ▶, 2008 ▶). For stacking intera­ctions, see: Hunter (1994 ▶). For hydrogen-bond motifs, see:, see: Bernstein *et al.* (1995 ▶); Etter (1990 ▶).
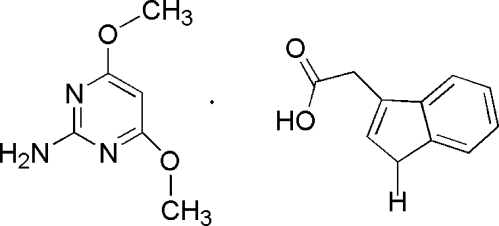


## Experimental

### 

#### Crystal data

C_10_H_9_NO_2_·C_6_H_9_N_3_O_2_
*M* *_r_* = 330.34
Triclinic, 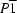

*a* = 7.4555 (1) Å
*b* = 10.7197 (2) Å
*c* = 11.2537 (2) Å
α = 62.981 (1)°
β = 85.863 (1)°
γ = 85.584 (1)°
*V* = 798.16 (2) Å^3^
*Z* = 2
Mo *K*α radiation
μ = 0.10 mm^−1^
*T* = 293 K
0.30 × 0.25 × 0.22 mm

#### Data collection

Bruker SMART APEXII CCD area-detector diffractometer
Absorption correction: multi-scan (*SADABS*; Bruker, 2008 ▶) *T*_min_ = 0.970, *T*_max_ = 0.978
19719 measured reflections
5363 independent reflections
3979 reflections with *I* > 2σ(*I*)
*R*_int_ = 0.028

#### Refinement

*R*[*F* ^2^ > 2σ(*F* ^2^)] = 0.046
*wR*(*F* ^2^) = 0.137
*S* = 1.06
5363 reflections
220 parameters
H-atom parameters constrained
Δρ_max_ = 0.23 e Å^−3^
Δρ_min_ = −0.22 e Å^−3^

### 

Data collection: *APEX2* (Bruker, 2008 ▶); cell refinement: *SAINT* (Bruker, 2008 ▶); data reduction: *SAINT*; program(s) used to solve structure: *SHELXS97* (Sheldrick, 2008 ▶); program(s) used to refine structure: *SHELXL97* (Sheldrick, 2008 ▶); molecular graphics: *PLATON* (Spek, 2009 ▶); software used to prepare material for publication: *PLATON*.

## Supplementary Material

Crystal structure: contains datablocks global, I. DOI: 10.1107/S1600536810037724/bv2155sup1.cif

Structure factors: contains datablocks I. DOI: 10.1107/S1600536810037724/bv2155Isup2.hkl

Additional supplementary materials:  crystallographic information; 3D view; checkCIF report

## Figures and Tables

**Table 1:** Hydrogen-bond geometry (Å, °)

*D*—H⋯*A*	*D*—H	H⋯*A*	*D*⋯*A*	*D*—H⋯*A*
N2—H2*A*⋯O4	0.86	2.04	2.8927 (14)	171
O3—H3⋯N1	0.82	1.88	2.6979 (12)	172
N4—H4⋯N3^i^	0.86	2.45	3.2184 (17)	149
C10—H10*A*⋯O4^ii^	0.97	2.59	3.5491 (18)	172

Symmetry codes: (i) 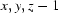; (ii) 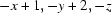.

**Fig. 1.**
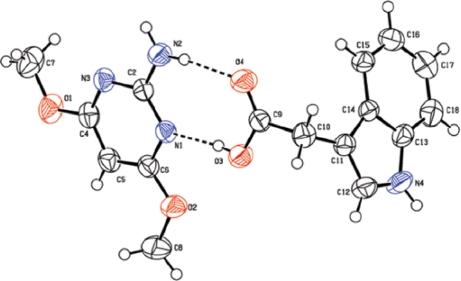
The asymmetric unit of (I), showing 50% probability displacement ellipsoids. Dashed lines indicate hydrogen bonds.

**Fig. 2.**
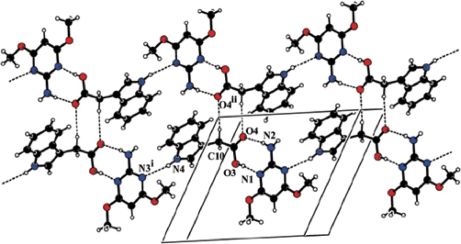
The crystal structure of (I). Dashed lines indicate hydrogen bonds H atoms not involved in hydrogen bonding have been omitted [symmetry codes: (i) x, y, z - 1; (ii) -x + 1, -y + 2, -z]

## supplementary crystallographic information

### Comment

A study of non-covalent interactions, such as hydrogen bonding, plays a key role in molecular recognition and crystal engineering (Desiraju, 1989). The prime importance of aminopyrimidine-carboxylate interactions is due to their involvement in protein-nuleic acid recognition and protein-drug binding (Hunt *et al.*, 1980; Baker & Santi, 1965). Aminopyrimidines readily pair up with carboxylic acids to form adducts rather than individual self-assembly compounds which is evident from the fact that 2-aminopyrimidine forms a wide variety of 1:1 adducts with mono and dicarboxylic acids (Etter & Adsmond, 1990) rather than individual self-assembly compounds (Scheinbeim & Schempp, 1976). The *R*_2_^2^(8) motif is a robust synthon which is frequently observed when a carboxylic acid interacts with a 2-amino heterocyclic ring system (Lynch & Jones, 2004). This motif is also recognized to be one of the top 5 motifs among the 24 commonly occurring motifs in crystal structures (Allen *et al.*, 1998). Auxin is a plant growth hormone which induces cell elongation in stems. 2-(1H-indol-3-yl)acetic acid is the first isolated auxin (Arteca, 1996). The crystal structures of 4,6-dimethoxypyrimidin-2-amine (Low *et al.*, 2002) and 2-(1H-indol-3-yl)acetic acid (Karle *et al.*,1964) have already been reported. Several cocrystals of 4,6-dimethoxypyrimidin-2-amine with various carboxylic acids such as 4,6-dimethoxypyrimidin-2-amine 4-aminobenzoic acid (1/1) (Thanigaimani *et al.*, 2006), 4,6-dimethoxypyrimidin-2-amine phthalic acid (1/1) (Thanigaimani *et al.*, 2007) and 4,6-dimethoxypyrimidin-2-amine anthranilic acid (1/1) (Thanigaimani *et al.*, 2008) have been recently reported from our group. In the present study, the various hydrogen-bonding patterns in the 4,6-dimethoxypyrimidin-2-amine (1H-indol-3-yl)acetic acid (1/1) cocrystal, (I), are thoroughly investigated.

The asymmetric unit (Fig. 1) contains a molecule of 4,6-dimethoxypyrimidin-2-amine and a molecule of 2-(1H-indol-3-yl)acetic acid, which are linked by N—H···O and O—H···N hydrogen bonds (Table. 1), forming an eight-membered ring with graph-set notation *R*_2_^2^(8) (Etter, 1990; Bernstein *et al.*, 1995). This motif is further linked by an N—H···N hydrogen bond, involving the N3 atom of 4,6-dimethoxypyrimidin-2-amine and N4 atom of the 2-(1H-indol-3-yl)acetic acid molecule, to form a supramolecular chain along the *c* axis. This supramolecular chain is further interlinked with a neighboring chain through a couple of C—H···O hydrogen bonds. These C—H···O hydrogen bonds form another *R*_2_^2^(8) motif. Further N—H···O, N—H···N and C—H···O hydrogen bonds combine together to form a large ring motif, with graph-set notation *R*_6_^4^(22). This ring motif extends to give a one dimensional supramolecular ladder as shown in Fig. 2. π-π stacking interaction is observed between two aminopyrimidine rings. They have an interplanar distance, centroid-to-centroid distance and a slip angle (the angle between the centroid vector and the normal to the plane) of 3.4413 (4) Å, 3.4937 (6) Å and 9.93° respectively. These are typical aromatic stacking values (Hunter, 1994).

### Experimental

A hot ethanolic solution (20 ml) of 4,6-dimethoxypyrimidin-2-amine (38 mg, Aldrich) and 2-(1H-indol-3-yl)acetic acid (44 mg, Loba Chemie) was warmed for half an hour over a water bath. The mixture was cooled slowly and kept at room temperature; afer a few days, colourless plate-like crystals were obtained.

### Refinement

All hydrogen atoms were positioned geometrically and were refined using a riding model. The N—H, O—H and C—H bond lengths are 0.86, 0.82 and 0.93–0.97 Å, respectively [*U*_iso_(H)=1.2 *U*_eq_ (parent atom)].

### Crystal data

**Table:**

C_10_H_9_NO_2_·C_6_H_9_N_3_O_2_	*Z* = 2
*M**_r_* = 330.34	*F*(000) = 348
Triclinic, *P*1	*D*_x_ = 1.375 Mg m^−^^3^
Hall symbol: -P 1	Mo *K*α radiation, λ = 0.71073 Å
*a* = 7.4555 (1) Å	Cell parameters from 5363 reflections
*b* = 10.7197 (2) Å	θ = 2.0–31.8°
*c* = 11.2537 (2) Å	µ = 0.10 mm^−^^1^
α = 62.981 (1)°	*T* = 293 K
β = 85.863 (1)°	Prism, colourless
γ = 85.584 (1)°	0.30 × 0.25 × 0.22 mm
*V* = 798.16 (2) Å^3^	

### Data collection

**Table:**

Bruker SMART APEXII CCD area-detector diffractometer	5363 independent reflections
Radiation source: fine-focus sealed tube	3979 reflections with *I* > 2σ(*I*)
graphite	*R*_int_ = 0.028
φ and ω scans	θ_max_ = 31.8°, θ_min_ = 2.0°
Absorption correction: multi-scan (*SADABS*; Bruker, 2008)	*h* = −10→10
*T*_min_ = 0.970, *T*_max_ = 0.978	*k* = −15→15
19719 measured reflections	*l* = −16→16

### Refinement

**Table:**

Refinement on *F*^2^	Primary atom site location: structure-invariant direct methods
Least-squares matrix: full	Secondary atom site location: difference Fourier map
*R*[*F*^2^ > 2σ(*F*^2^)] = 0.046	Hydrogen site location: inferred from neighbouring sites
*wR*(*F*^2^) = 0.137	H-atom parameters constrained
*S* = 1.06	*w* = 1/[σ^2^(*F*_o_^2^) + (0.0654*P*)^2^ + 0.0927*P*] where *P* = (*F*_o_^2^ + 2*F*_c_^2^)/3
5363 reflections	(Δ/σ)_max_ < 0.001
220 parameters	Δρ_max_ = 0.23 e Å^−^^3^
0 restraints	Δρ_min_ = −0.22 e Å^−^^3^

### Special details

**Table:**

Geometry. Bond distances, angles *etc*. have been calculated using the rounded fractional coordinates. All su's are estimated from the variances of the (full) variance-covariance matrix. The cell e.s.d.'s are taken into account in the estimation of distances, angles and torsion angles
Refinement. Refinement on *F*^2^ for ALL reflections except those flagged by the user for potential systematic errors. Weighted *R*-factors *wR* and all goodnesses of fit *S* are based on *F*^2^, conventional *R*-factors *R* are based on *F*, with *F* set to zero for negative *F*^2^. The observed criterion of *F*^2^ > σ(*F*^2^) is used only for calculating -*R*-factor-obs *etc*. and is not relevant to the choice of reflections for refinement. *R*-factors based on *F*^2^ are statistically about twice as large as those based on *F*, and *R*-factors based on ALL data will be even larger.

### Fractional atomic coordinates and isotropic or equivalent isotropic displacement parameters (Å^2^)

**Table:**

	*x*	*y*	*z*	*U*_iso_*/*U*_eq_	
O3	0.47314 (13)	0.61367 (8)	0.19523 (8)	0.0511 (3)	
O4	0.41360 (15)	0.83007 (9)	0.17016 (9)	0.0610 (3)	
N4	0.33691 (16)	0.67797 (12)	−0.21918 (11)	0.0549 (4)	
C9	0.47507 (14)	0.75081 (11)	0.12695 (11)	0.0401 (3)	
C10	0.56115 (15)	0.79980 (13)	−0.01155 (11)	0.0458 (3)	
C11	0.44583 (15)	0.77265 (11)	−0.09994 (11)	0.0409 (3)	
C12	0.47745 (18)	0.67593 (13)	−0.14708 (13)	0.0514 (4)	
C13	0.20837 (16)	0.77659 (11)	−0.21974 (11)	0.0429 (3)	
C14	0.27307 (14)	0.83893 (10)	−0.14517 (10)	0.0372 (3)	
C15	0.16352 (16)	0.94084 (11)	−0.12571 (11)	0.0438 (3)	
C16	−0.00356 (18)	0.97665 (13)	−0.17950 (13)	0.0526 (4)	
C17	−0.06486 (18)	0.91367 (14)	−0.25289 (14)	0.0567 (4)	
C18	0.03945 (18)	0.81345 (14)	−0.27433 (13)	0.0532 (4)	
O1	0.05798 (14)	0.34071 (10)	0.80461 (9)	0.0602 (3)	
O2	0.34975 (14)	0.31388 (9)	0.43080 (9)	0.0556 (3)	
N1	0.29633 (12)	0.51850 (9)	0.43555 (8)	0.0385 (2)	
N2	0.24541 (15)	0.73208 (10)	0.43481 (10)	0.0492 (3)	
N3	0.15054 (12)	0.54001 (10)	0.62253 (9)	0.0421 (3)	
C2	0.23037 (13)	0.59256 (10)	0.49907 (10)	0.0368 (3)	
C4	0.13751 (15)	0.40253 (12)	0.68250 (11)	0.0433 (3)	
C5	0.20213 (16)	0.31430 (11)	0.62803 (11)	0.0450 (3)	
C6	0.28114 (14)	0.37938 (11)	0.50225 (11)	0.0398 (3)	
C7	−0.0202 (2)	0.42776 (17)	0.86200 (15)	0.0684 (5)	
C8	0.3549 (2)	0.16378 (14)	0.49465 (17)	0.0659 (5)	
H3	0.41670	0.59300	0.26640	0.0770*	
H4	0.33010	0.62570	−0.25810	0.0660*	
H10A	0.57970	0.89940	−0.05000	0.0550*	
H10B	0.67780	0.75120	−0.00610	0.0550*	
H12	0.58030	0.61690	−0.13220	0.0620*	
H15	0.20310	0.98350	−0.07720	0.0530*	
H16	−0.07720	1.04400	−0.16680	0.0630*	
H17	−0.17860	0.94000	−0.28800	0.0680*	
H18	−0.00150	0.77200	−0.32350	0.0640*	
H2A	0.29580	0.77020	0.35610	0.0590*	
H2B	0.20460	0.78330	0.47240	0.0590*	
H5	0.19270	0.21760	0.67350	0.0540*	
H7A	0.07320	0.47350	0.87930	0.1030*	
H7B	−0.08420	0.37130	0.94420	0.1030*	
H7C	−0.10200	0.49700	0.80110	0.1030*	
H8A	0.42070	0.12850	0.57470	0.0990*	
H8B	0.41310	0.12980	0.43540	0.0990*	
H8C	0.23430	0.13240	0.51670	0.0990*	

### Atomic displacement parameters (Å^2^)

**Table:**

	*U*^11^	*U*^22^	*U*^33^	*U*^12^	*U*^13^	*U*^23^
O3	0.0697 (5)	0.0377 (4)	0.0429 (4)	−0.0047 (4)	0.0080 (4)	−0.0168 (3)
O4	0.0885 (7)	0.0381 (4)	0.0480 (5)	0.0014 (4)	0.0132 (5)	−0.0150 (4)
N4	0.0687 (7)	0.0542 (6)	0.0561 (6)	0.0007 (5)	0.0025 (5)	−0.0387 (5)
C9	0.0420 (5)	0.0379 (5)	0.0382 (5)	−0.0017 (4)	−0.0039 (4)	−0.0151 (4)
C10	0.0439 (5)	0.0476 (6)	0.0420 (6)	−0.0072 (4)	0.0042 (4)	−0.0170 (5)
C11	0.0467 (5)	0.0377 (5)	0.0352 (5)	−0.0028 (4)	0.0065 (4)	−0.0149 (4)
C12	0.0560 (6)	0.0490 (6)	0.0510 (7)	0.0056 (5)	0.0053 (5)	−0.0265 (5)
C13	0.0552 (6)	0.0392 (5)	0.0349 (5)	−0.0069 (4)	0.0053 (4)	−0.0175 (4)
C14	0.0473 (5)	0.0311 (4)	0.0300 (4)	−0.0056 (4)	0.0055 (4)	−0.0115 (4)
C15	0.0564 (6)	0.0341 (5)	0.0398 (5)	−0.0021 (4)	0.0030 (4)	−0.0165 (4)
C16	0.0553 (6)	0.0429 (6)	0.0527 (7)	0.0058 (5)	0.0020 (5)	−0.0174 (5)
C17	0.0521 (6)	0.0554 (7)	0.0534 (7)	−0.0018 (5)	−0.0064 (5)	−0.0161 (6)
C18	0.0626 (7)	0.0539 (7)	0.0441 (6)	−0.0107 (6)	−0.0034 (5)	−0.0216 (5)
O1	0.0812 (6)	0.0527 (5)	0.0390 (4)	−0.0194 (4)	0.0135 (4)	−0.0137 (4)
O2	0.0780 (6)	0.0368 (4)	0.0509 (5)	−0.0012 (4)	0.0043 (4)	−0.0201 (4)
N1	0.0450 (4)	0.0338 (4)	0.0330 (4)	−0.0032 (3)	−0.0023 (3)	−0.0117 (3)
N2	0.0683 (6)	0.0343 (5)	0.0418 (5)	−0.0048 (4)	0.0059 (4)	−0.0152 (4)
N3	0.0480 (5)	0.0405 (5)	0.0349 (4)	−0.0054 (4)	−0.0001 (3)	−0.0142 (4)
C2	0.0388 (5)	0.0352 (5)	0.0341 (5)	−0.0026 (4)	−0.0056 (4)	−0.0129 (4)
C4	0.0469 (5)	0.0443 (6)	0.0335 (5)	−0.0096 (4)	−0.0026 (4)	−0.0119 (4)
C5	0.0556 (6)	0.0342 (5)	0.0389 (5)	−0.0082 (4)	−0.0046 (4)	−0.0098 (4)
C6	0.0454 (5)	0.0348 (5)	0.0378 (5)	−0.0026 (4)	−0.0065 (4)	−0.0145 (4)
C7	0.0834 (10)	0.0718 (9)	0.0483 (7)	−0.0218 (7)	0.0227 (7)	−0.0266 (7)
C8	0.0883 (10)	0.0380 (6)	0.0730 (9)	−0.0021 (6)	−0.0022 (8)	−0.0268 (6)

### Geometric parameters (Å, °)

**Table:**

O3—C9	1.3142 (15)	C13—C18	1.3907 (18)
O4—C9	1.2048 (16)	C14—C15	1.4009 (17)
O3—H3	0.8200	C15—C16	1.3746 (18)
O1—C7	1.427 (2)	C16—C17	1.398 (2)
O1—C4	1.3397 (14)	C17—C18	1.377 (2)
O2—C6	1.3415 (16)	C10—H10A	0.9700
O2—C8	1.432 (2)	C10—H10B	0.9700
N4—C12	1.3637 (18)	C12—H12	0.9300
N4—C13	1.3693 (18)	C15—H15	0.9300
N4—H4	0.8600	C16—H16	0.9300
N1—C2	1.3371 (15)	C17—H17	0.9300
N1—C6	1.3405 (16)	C18—H18	0.9300
N2—C2	1.3431 (16)	C4—C5	1.3843 (18)
N3—C2	1.3502 (13)	C5—C6	1.3723 (16)
N3—C4	1.3213 (17)	C5—H5	0.9300
N2—H2B	0.8600	C7—H7A	0.9600
N2—H2A	0.8600	C7—H7B	0.9600
C9—C10	1.5103 (16)	C7—H7C	0.9600
C10—C11	1.4961 (17)	C8—H8A	0.9600
C11—C14	1.4311 (16)	C8—H8B	0.9600
C11—C12	1.362 (2)	C8—H8C	0.9600
C13—C14	1.4146 (17)		
			
O2···C12^i^	3.3132 (16)	C5···H8C	2.7400
O2···N4^i^	3.1900 (15)	C5···H8A	2.7300
O3···N4^i^	3.2341 (17)	C6···H3	2.7900
O3···N1	2.6979 (12)	C8···H5	2.5400
O3···C12^i^	3.3749 (18)	C9···H2A	2.9000
O3···C4^ii^	3.2606 (15)	C12···H8B^i^	3.0400
O4···N2	2.8927 (14)	C13···H7B^vii^	2.8900
O1···H10B^ii^	2.8800	C14···H7B^vii^	2.7500
O2···H3	2.7700	C15···H5^viii^	2.8100
O2···H4^i^	2.8800	C16···H5^viii^	2.8100
O3···H4^i^	2.6800	H2A···C9	2.9000
O4···H2A	2.0400	H2A···O4	2.0400
O4···H10A^iii^	2.5900	H3···N1	1.8800
O4···H16^iv^	2.7500	H3···C2	2.8700
N1···O3	2.6979 (12)	H3···C6	2.7900
N2···O4	2.8927 (14)	H3···O2	2.7700
N3···N4^v^	3.2184 (17)	H4···O3^i^	2.6800
N4···O3^i^	3.2341 (17)	H4···C2^vi^	3.0600
N4···N3^vi^	3.2184 (17)	H4···H7A^vi^	2.5500
N4···O2^i^	3.1900 (15)	H4···N3^vi^	2.4500
N1···H3	1.8800	H4···O2^i^	2.8800
N3···H18^v^	2.9500	H5···C15^ix^	2.8100
N3···H7C	2.5600	H5···C16^ix^	2.8100
N3···H4^v^	2.4500	H5···C8	2.5400
N3···H7A	2.6700	H5···H8A	2.3400
N4···H7A^vi^	2.8400	H5···H8C	2.3200
C2···C4^vii^	3.5198 (15)	H7A···N4^v^	2.8400
C2···C5^vii^	3.5089 (15)	H7A···N3	2.6700
C4···C9^ii^	3.5501 (16)	H7A···H4^v^	2.5500
C4···O3^ii^	3.2606 (15)	H7B···C13^vii^	2.8900
C4···C2^vii^	3.5198 (15)	H7B···C14^vii^	2.7500
C5···C2^vii^	3.5089 (15)	H7C···N3	2.5600
C5···C9^ii^	3.5822 (16)	H8A···H5	2.3400
C9···C15	3.5484 (16)	H8A···C5	2.7300
C9···C4^ii^	3.5501 (16)	H8B···C12^i^	3.0400
C9···C5^ii^	3.5822 (16)	H8C···H5	2.3200
C12···O3^i^	3.3749 (18)	H8C···C5	2.7400
C12···O2^i^	3.3132 (16)	H10A···O4^iii^	2.5900
C15···C9	3.5484 (16)	H10B···O1^ii^	2.8800
C2···H3	2.8700	H16···O4^iv^	2.7500
C2···H4^v^	3.0600	H18···N3^vi^	2.9500
			
C9—O3—H3	109.00	C11—C12—H12	125.00
C4—O1—C7	118.24 (12)	N4—C12—H12	125.00
C6—O2—C8	117.57 (11)	C16—C15—H15	121.00
C12—N4—C13	109.14 (12)	C14—C15—H15	121.00
C12—N4—H4	125.00	C17—C16—H16	119.00
C13—N4—H4	125.00	C15—C16—H16	119.00
C2—N1—C6	116.09 (9)	C18—C17—H17	119.00
C2—N3—C4	115.07 (11)	C16—C17—H17	119.00
C2—N2—H2B	120.00	C13—C18—H18	121.00
C2—N2—H2A	120.00	C17—C18—H18	121.00
H2A—N2—H2B	120.00	N1—C2—N2	117.23 (9)
O3—C9—C10	113.48 (11)	N1—C2—N3	126.02 (11)
O4—C9—C10	123.11 (12)	N2—C2—N3	116.74 (11)
O3—C9—O4	123.41 (11)	N3—C4—C5	124.42 (10)
C9—C10—C11	111.17 (10)	O1—C4—N3	119.52 (12)
C12—C11—C14	106.25 (11)	O1—C4—C5	116.06 (12)
C10—C11—C14	126.02 (11)	C4—C5—C6	115.35 (11)
C10—C11—C12	127.64 (11)	O2—C6—N1	111.97 (10)
N4—C12—C11	110.41 (12)	O2—C6—C5	124.99 (12)
N4—C13—C14	107.17 (10)	N1—C6—C5	123.04 (11)
C14—C13—C18	122.00 (12)	C4—C5—H5	122.00
N4—C13—C18	130.78 (13)	C6—C5—H5	122.00
C11—C14—C13	107.03 (10)	O1—C7—H7A	109.00
C11—C14—C15	133.95 (11)	O1—C7—H7B	109.00
C13—C14—C15	118.96 (10)	O1—C7—H7C	109.00
C14—C15—C16	118.84 (12)	H7A—C7—H7B	109.00
C15—C16—C17	121.25 (13)	H7A—C7—H7C	109.00
C16—C17—C18	121.46 (13)	H7B—C7—H7C	110.00
C13—C18—C17	117.48 (13)	O2—C8—H8A	110.00
H10A—C10—H10B	108.00	O2—C8—H8B	109.00
C11—C10—H10B	109.00	O2—C8—H8C	109.00
C11—C10—H10A	109.00	H8A—C8—H8B	109.00
C9—C10—H10A	109.00	H8A—C8—H8C	109.00
C9—C10—H10B	109.00	H8B—C8—H8C	109.00
			
C7—O1—C4—C5	−176.42 (11)	C10—C11—C12—N4	176.92 (11)
C7—O1—C4—N3	3.89 (17)	C10—C11—C14—C13	−176.77 (11)
C8—O2—C6—N1	175.18 (11)	C10—C11—C14—C15	0.2 (2)
C8—O2—C6—C5	−5.60 (17)	C12—C11—C14—C13	−0.14 (13)
C13—N4—C12—C11	−0.47 (15)	C12—C11—C14—C15	176.84 (13)
C12—N4—C13—C14	0.36 (14)	N4—C13—C14—C15	−177.65 (10)
C12—N4—C13—C18	−177.05 (13)	C18—C13—C14—C11	177.55 (11)
C6—N1—C2—N3	−0.22 (15)	N4—C13—C14—C11	−0.13 (13)
C2—N1—C6—O2	179.64 (9)	C18—C13—C14—C15	0.03 (17)
C6—N1—C2—N2	179.42 (10)	N4—C13—C18—C17	176.85 (13)
C2—N1—C6—C5	0.40 (16)	C14—C13—C18—C17	−0.23 (19)
C2—N3—C4—C5	1.29 (16)	C13—C14—C15—C16	0.16 (16)
C4—N3—C2—N2	179.77 (10)	C11—C14—C15—C16	−176.54 (12)
C2—N3—C4—O1	−179.05 (10)	C14—C15—C16—C17	−0.16 (19)
C4—N3—C2—N1	−0.59 (15)	C15—C16—C17—C18	0.0 (2)
O4—C9—C10—C11	−109.62 (14)	C16—C17—C18—C13	0.2 (2)
O3—C9—C10—C11	70.10 (13)	O1—C4—C5—C6	179.20 (10)
C9—C10—C11—C12	−109.00 (14)	N3—C4—C5—C6	−1.13 (17)
C9—C10—C11—C14	66.91 (16)	C4—C5—C6—O2	−178.92 (11)
C14—C11—C12—N4	0.37 (14)	C4—C5—C6—N1	0.21 (17)

Symmetry codes: (i) −*x*+1, −*y*+1, −*z*; (ii) −*x*+1, −*y*+1, −*z*+1; (iii) −*x*+1, −*y*+2, −*z*; (iv) −*x*, −*y*+2, −*z*; (v) *x*, *y*, *z*+1; (vi) *x*, *y*, *z*−1; (vii) −*x*, −*y*+1, −*z*+1; (viii) *x*, *y*+1, *z*−1; (ix) *x*, *y*−1, *z*+1.

### Hydrogen-bond geometry (Å, °)

**Table:**

*D*—H···*A*	*D*—H	H···*A*	*D*···*A*	*D*—H···*A*
N2—H2A···O4	0.86	2.04	2.8927 (14)	171
O3—H3···N1	0.82	1.88	2.6979 (12)	172
N4—H4···N3^vi^	0.86	2.45	3.2184 (17)	149
C10—H10A···O4^iii^	0.97	2.59	3.5491 (18)	172

Symmetry codes: (vi) *x*, *y*, *z*−1; (iii) −*x*+1, −*y*+2, −*z*.
